# New insights into the genic and metabolic characteristics of induced pluripotent stem cells from polycystic ovary syndrome women

**DOI:** 10.1186/s13287-018-0950-x

**Published:** 2018-08-09

**Authors:** Zheying Min, Qian Gao, Xiumei Zhen, Yong Fan, Tao Tan, Rong Li, Yue Zhao, Yang Yu

**Affiliations:** 1 Department of Obstetrics and Gynecology, Beijing Key Laboratory of Reproductive Endocrinology and Assisted Reproductive Technology and Key Laboratory of Assisted Reproduction, Ministry of Education, Center for Reproductive Medicine, Peking University Third Hospital, Beijing, 100191 China; 2 Peking-Tsinghua Center for Life Sciences, Peking University, Beijing, 100871 China; 3 Key Laboratory for Major Obstetric Diseases of Guangdong Province, The Third Affiliated Hospital of Guangzhou Medical University, Guangzhou, 510150 China; 4 Yunnan Key Laboratory of Primate Biomedical Research, Institute of Primate Translational Medicine, Kunming University of Science and Technology, Kunming, 650500 Yunnan China

**Keywords:** PCOS, Induced pluripotent stem cells, RNA microarray, Mitochondria, Metabolism

## Abstract

**Background:**

Polycystic ovary syndrome (PCOS) is a common endocrine and metabolic disorder that affects female fertility. However, with the lack of a corresponding research model, the pathology mechanism of PCOS is poorly understood. Induced pluripotent stem cell (iPSC) technology has been recognized as means to generate patient-specific stem cells for disease modeling.

**Methods:**

The mRNA abundance of iPSCs was analyzed by RNA microarray and real-time polymerase chain reaction (RT-PCR). Karyotyping of iPSCs was performed with cytogenetic analysis. The mitochondrial respiration ability and glycolytic function were measured by the Seahorse Bioscience XF extracellular flux analyzer. The expression of iPSC-associated markers was identified by immunofluorescence and RT-PCR. The teratoma formation of iPSCs was studied using immunochemistry.

**Results:**

A PCOS patient-derived iPSC model was established from somatic cells of PCOS patients. Through comprehensive transcriptional profiling analysis of the RNA microarray, PCOS patient-derived iPSCs showed metabolic abnormalities and mitochondrial dysfunction compared with non-PCOS patient-derived iPSCs in vitro﻿. Specifically, a total of 2904 genes were differentially expressed between the two iPSC populations, of which 1416 genes were upregulated and 1488 genes were downregulated (fold change > 2, *p* < 0.01). Gene Ontology (GO) term enrichment results showed that upregulated genes were enriched in metabolic processes and mitochondrial activities which participated in the tricarboxylic acid (TCA) cycle, the respiratory electron transport chain (ETC), and glycogenolysis. On the other hand, the downregulated genes were related to cell communication, glucose transport, and uptake. The differentially expressed genes were verified by RT-PCR in PCOS patient-derived iPSCs and granulosa cells from PCOS patients. The PCOS patient-derived iPSCs demonstrated decreased mitochondrial respiration ability and glycolytic function (*p* < 0.05) but increased mitochondrial copy numbers and biogenesis (*p* < 0.05). Subsequently, some genes related to glucose metabolism were rescued by treating with metformin in PCOS patient-derived iPSCs. Meanwhile, the ATP production ability of mitochondria and the glycolysis ability of PCOS patient-derived iPSCs also partially returned to normal levels. However, metformin had little effect on mitochondrial maximal respiration ability and maximal glycolytic capacity.

**Conclusions:**

We measured differences in iPSCs from women with and without PCOS in gene transcription and mitochondrial respiratory function. PCOS patient-derived iPSCs showed abnormal expression of metabolic genes and mitochondrial dysfunction in vitro*.* The study provides a novel cell model in vitro for studying the clinical causes and molecular mechanisms of PCOS.

**Electronic supplementary material:**

The online version of this article (10.1186/s13287-018-0950-x) contains supplementary material, which is available to authorized users.

## Background

Polycystic ovary syndrome (PCOS) is a common endocrine and metabolic disorder that affects female fertility, with an incidence of 5% to 10% [1]. Hyperandrogenism, oligomenorrhea, chronic anovulation, and hyperinsulinemia from insulin resistance (IR) are classic clinical manifestations of PCOS [2]. Recent studies have indicated that PCOS is also associated with cardiovascular disease (CVD), lipid metabolism disorder, and type 2 diabetes mellitus (DM2) [3]. The clinical cause and molecular mechanism of PCOS remain unclear, although it is considered a polygenic pathology that might result from the interaction of susceptible genomic variants and environmental factors [4, 5]. Therefore, further understanding of this disease is required to determine the pathogenesis of PCOS and to develop new strategies to treat PCOS efficiently.

Metabolic disorders have been acknowledged as a frequent cause of classic PCOS [6]. At present, changes in several metabolic pathways have been implicated in the pathophysiology of PCOS, including abnormalities in steroid hormone regulation and insulin signaling [7–9]. Additionally, there is increasing attention on the complications related to metabolic disturbance among PCOS patients, such as obesity, IR, dyslipidemia, and inflammation, which have been recognized as risk factors for DM2 and CVD in PCOS [10–12]. Metabolism is crucial for cellular processes, and inefficiency in adjusting to variations in energy demand can disturb energy metabolism, such as the tricarboxylic acid (TCA) cycle and lipid or amino acid processing pathways [13]. Metabolism studies on DM2, obesity, and IR have detected changes in glucose metabolism, branched-chain amino acid catabolism, and fatty acid oxidation [12, 14]. This indicates the need to understand the metabolic abnormalities in PCOS to prevent complications through efficient screening, diagnosis, and intervention [15].

Metformin, an insulin sensitizer, has been introduced as a pharmaceutical option to target not only IR but also some other syndromes, including reproductive abnormalities [16]. Metformin is a potent antihyperglycemic agent used in type 2 diabetes and also used in PCOS treatment [17]. The biguanide metformin has pleiotropic effects on several tissues, and various mechanisms are involved in its inhibition of gluconeogenesis [18]. Potential mechanisms of metformin include direct inhibition of gluconeogenic enzymes (e.g., FBP1 and G6PC), increased uptake of substrates for gluconeogenesis, increased insulin receptor sensitivity, and inhibition of mitochondrial respiration to reduce the energy required for gluconeogenesis [19].

Furthermore, the phenomenon of PCOS familial aggregation implies that genetic factors play an important role in its etiology [20]. Some researchers consider that the pathological alterations begin during the embryonic stage and that spatial and temporal regulations play a role in the development of PCOS [21]. However, because of ethical and legal restrictions for human embryo studies and limited access to embryos with inherited PCOS, no appropriate inheritance model exists for PCOS, including no cellular model with a family history of and genetic tendency for PCOS. At present, there are only animal models that employ experimentally induced excess androgen to permanently induce PCOS-like metabolic and reproductive traits [22, 23]. For a dynamic and continuous developmental study in PCOS, an effective and convenient experimental cell model is necessary to explore PCOS pathogenesis.

Current studies in cellular reprogramming use induced pluripotent stem cells (iPSC) generated from adult cells to create patient-specific stem cells for exploring disease mechanisms and developing targeted therapies. iPSCs derived from the somatic cells of various disorders have been applied in disease models, such as diabetes mellitus [24, 25], Rett syndrome [26], and spinal muscle atrophy [27]. Most iPSCs demonstrate observable disease-specific phenotypes when differentiated into relevant cell types.

Here, iPSC lines derived from the somatic cells of classic PCOS patients were generated. We investigated the transcriptional profiles of these iPSC lines using RNA microarray and metabolic ability and studied the mitochondrial function of the PCOS patient-derived iPSCs in vitro. Subsequently, to evaluate the potential use of PCOS patient-derived iPSCs for drug discovery and further clinical therapy, metformin was used to demonstrate feedback in the PCOS patient-derived iPSC model. The PCOS patient-derived iPSCs provide a new biological cell model to study the pathogenesis of PCOS and to help discover new drugs for clinical therapies.

## Methods

### Ethics

The study was approved by Institutional Review Board and Ethics Committee of Peking University Third Hospital. Written informed consent was obtained from all patients enrolled in this study.

### Diagnostic criteria and characteristics of PCOS patients

The characteristics of the selected three PCOS and three non-PCOS women are shown in Table 1. In this study, subjects who had two of the following three conditions were diagnosed with PCOS [2]: 1) oligo or anovulation; 2) hyperandrogenism or clinical manifestations of hyperandrogenism, such as hirsutism and acne; 3) polycystic ovaries on ultrasonography with exclusion of related disorders, such as thyroid disease, congenital or atypical adrenal hyperplasia, and exogenous androgen application.

**Table 1:** Clinical parameter in women with polycystic ovary syndrome (PCOS) and non-PCOS women

Parameters	PCOS (patient ID)			non-PCOS (patient ID)			*P* value
	1	2	3	1	2	3	
Age at surgery (years)	33	30	33	29	31	31	ns
BMI (kg/m^2^)	24.9	27.3	25	23.2	22	22.9	ns
Cycle length (days)	120	180	180	30	29	28	**0.012**
FSH (mIU/ml)	4.73	5.62	6.3	8.37	8.95	7.22	**0.046**
LH (mIU/ml)	14.9	16.3	23.7	3.36	6.29	5.51	**0.017**
Estradiol (pmol/l)	400	339	335	156	177	114	**0.007**
Progesterone (nmol/l)	1.6	1.0	7.7	0.8	0.7	0.7	ns
Testosterone (nmol/l))	1.27	2.48	1.22	0.69	0.69	0.69	ns
Androstenedione (nmol/l)	12.5	11.2	17	3.6	4.9	4.2	**0.019**
Glucose (mmol/l)	5.4	4.8	5.2	4.92	5	4.7	ns
Insulin (mU/l)	29.1	14.6	18.7	–			

*P* values > 0.05 were considered nonsignificant (ns); *P* values < 0.05 are shown in bold

*BMI* body mass index, *FSH* follicle stimulating hormone, *LH* luteinizing hormone

### Derivation of primary fibroblast cells

Human dermal fibroblasts were derived from skin cells through operative incision. The skin tissues were digested into cell aggregates and cultured on Matrigel-coated dishes with Dulbecco’s modified Eagle’s medium (DMEM; Gibco, New York, USA) containing 10% (v/v) fetal bovine serum (FBS; HyClone, USA).

### Generation and culture of PCOS patient-derived iPSCs

The iPSC clones were reprogrammed from the fibroblast cells as described previously [28]. Briefly, 10% confluent epithelial cells were transduced with *OCT4*-, *SOX2*-, *KLF4*-, and *C-MYC*-expressing lentiviral vectors in mouse embryonic fibroblast (MEF) medium without serum. After overnight viral incubation, infected cells were seeded onto mitomycin C-treated MEFs. The medium was replaced with fresh complete MEF medium each day. On day 5, the medium was replaced with iPSC medium (DMEM/F12 supplemented with 20% (v/v) knockout serum replacer (Knockout SR), 2 mM l-glutamine, 2 mM nonessential amino acids, and 0.1 mM β-mercaptoethanol (all from Gibco, New York, USA) with no additional human basic fibroblast growth factor (bFGF)). Cells were cultured with fresh iPSC medium every day. Putative PCOS patient-derived iPSC colonies were observed within 21 days after infection. Generated colonies were mechanically dissociated. Dissociated cell clumps were plated into new wells with feeder cells for expansion.

### Teratoma formation

Approximately 5 × 10^6^ iPSCs of all PCOS- and non-PCOS patient-derived cells were collected and injected into the leg of 6- to 8-week-old nonobese/severe combined immunodeficiency mice. After 2 months, the xenografts were collected for histological analysis by hematoxylin and eosin (H&E) staining. Antihuman nuclein antibody was used for immunochemistry staining.

### Immunofluorescence staining and alkaline phosphatase activity

For immunofluorescence staining, cells were fixed in 4% (w/v) paraformaldehyde in phosphate-buffered saline (PBS) for 15 min at room temperature and then blocked with PBS containing 10% (w/v) bovine serum albumin (BSA) and 1% (v/v) Triton X-100 (Sigma-Aldrich) for 1 h at room temperature. After blocking, the cells were incubated with primary antibodies overnight at 4 °C, followed by incubation with secondary antibodies at room temperature. The primary antibodies (all at 1:200 dilution and from Abcam) were used to detect OCT4, SOX2, NANOG, and SSEA-1 expression. We visualized antigen localization using goat anti-mouse/rabbit Alexa Fluor 488, 555, and 594. The nuclei were stained with Hochest 33,342 (Sigma) at a final concentration of 0.01 mg/mL for 10 min. We detected alkaline phosphatase (ALP) activity using substrates (Beyotime) according to the protocol.

### Microarray

Microarray hybridization was carried out at CapitalBio (Beijing, China). Total RNA (100 ng) was used to prepare twice-amplified and labeled RNA for hybridization with HG-UI33 plus 2.0 arrays. Each group contained three iPSC lines of patients. Each sample was processed individually on a separate RNA microarray chip in two runs.

### Quantitative real-time polymerase chain reaction (RT-PCR)

Total RNA was extracted from iPSCs using Trizol reagent (Invitrogen), and cDNA was synthesized in a 20-μl reaction using a ReverAid First Strand cDNA Synthesis Kit (Invitrogen). RNA was reverse-transcribed in a thermocycler using reverse transcriptase according to the manufacturer’s protocol. PCR amplification for different genes was performed using the SYBR Green mix kit (Invitrogen). The PCR products were amplified using an ABI7500 machine (Applied Biosystems, USA). Fold-change by RT-PCR was measured and calculated after normalizing to the housekeeping gene (β-actin) and calculating the average comparative threshold cycle (ΔC_t_). Statistical analyses were conducted by two sample *t* test. Differences were considered to be statistically significant when *P* < 0.05. Additional file 1: Table S1 lists the primers used.

### Mitochondrial oxygen consumption detection and glycolysis function test

The XF24 extracellular flux analyzer from Seahorse Bioscience (Billerica, MA, USA) was used to assess mitochondrial respiration and energy production in iPSCs. Oxygen consumption rates (OCRs) were measured in real time in PCOS- and non-PCOS patient-derived iPSCs. A classical Mito stress test was performed based on the following procedure: 1) the basal respiration was measured in unbuffered medium; 2) oligomycin (2.0 μM final concentration), an inhibitor of ATP production, was added; 3) the uncoupler carbonyl cyanide 4-(trifluoromethoxy) phenylhydrazone (FCCP; 2.0 μM final concentration) was added to measure maximal respiration; and 4) rotenone and antimycin A (0.5 μM final concentrations) were applied in combination to block respiration due to simultaneous inhibition of complexes I and III, respectively. An optimal cell density of 10^5^ cells per well was determined experimentally. The results were normalized to cell number and analyzed using Seahorse XF24 software.

A classical glycolysis stress test was performed based on the following procedure: 1) cells were cultured in unbuffered medium without glucose and pyruvate; 2) the extracellular acidification rate (ECAR) was measured after the addition of saturating amounts of glucose; 3) oligomycin was added to shut down oxidative phosphorylation; and 4) 2-DG was added to inhibit glycolysis.

### Identification of mitochondrial DNA (mtDNA) content in iPSCs

Total DNA was extracted from iPSCs using the QIAamp kit (Qiagen, Crawley, UK) according to the manufacturer’s instructions. Relative amounts of nuclear DNA (nDNA) and mtDNA were determined by RT-PCR. mtDNA was quantified using primers D41 and D56, as reported in a previous study [29], based on the relative cycle threshold (ΔΔC_t_) normalized to the control. nDNA was quantified according to actin gene expression.

### Statistical analysis

The microarray data were analyzed to identify statistically significantly different expression between PCOS- and non-PCOS patient-derived iPSCs. The list of identified genes (fold-change (FC) > 2; false discovery rate (FDR) < 0.05) was submitted to AmiGO2 (http://amigo.geneontology.org) to identify the Gene Ontology (GO) terms associated with biological functions and pathways. Data are presented as the mean ± standard deviation (SD) and were analyzed using the GraphPad Prism 5 program (GraphPad Software, San Diego, CA, USA).

## Results

### Generation of PCOS patient-derived iPSCs from adult cells

**Fig. 1:**
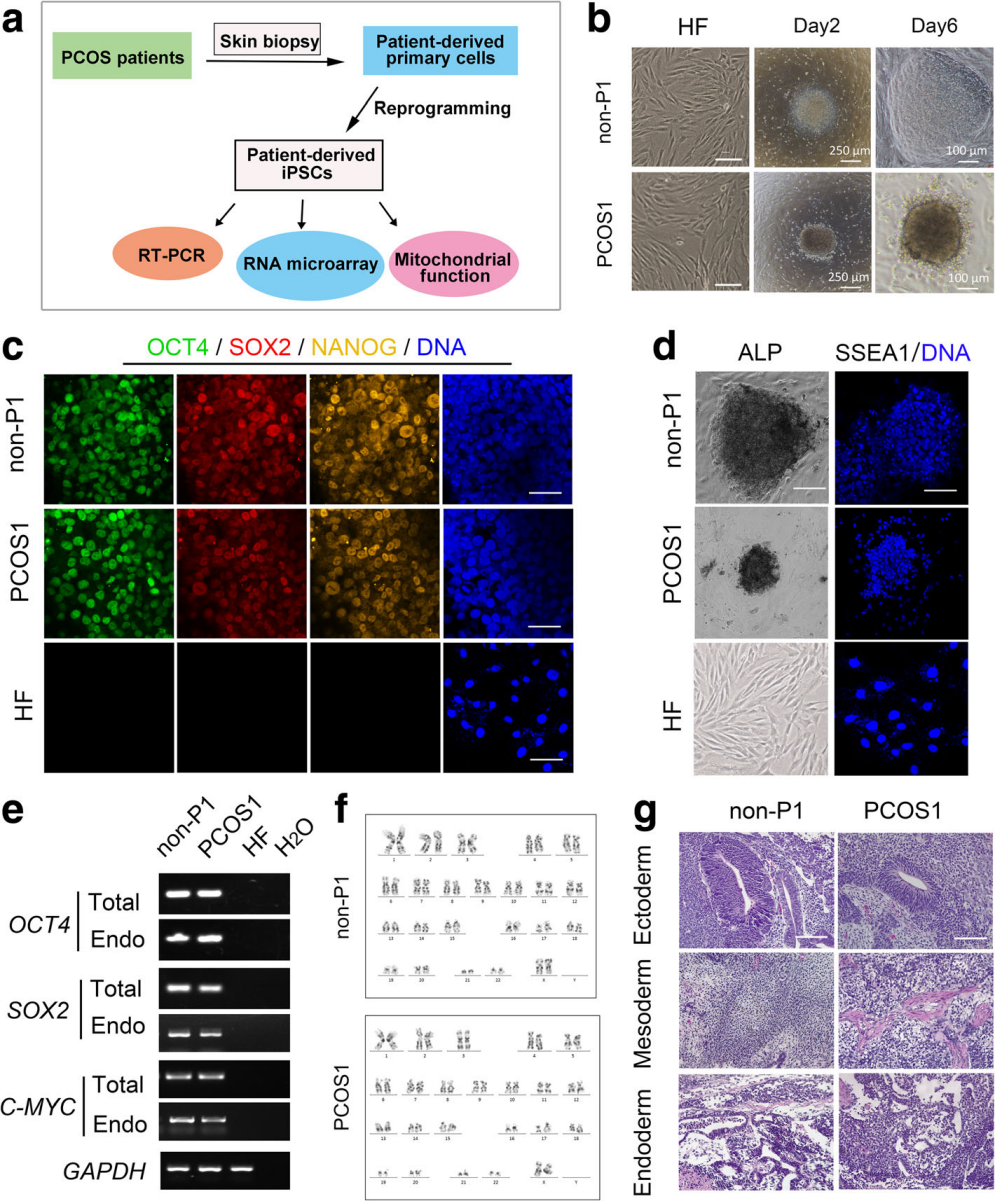
The phenotype and characteristics of PCOS patient-derived and non-PCOS patient-derived iPSCs. **a** Polycystic ovary syndrome (PCOS) disease modeling using induced pluripotent stem cell (iPSC) technology. After reprogramming, total RNA from PCOS patient-derived iPSCs was extracted for RNA microarray and quantitative real-time polymerase chain reaction (RT-PCR). Then, the mitochondrial functions of PCOS patient-derived iPSCs were measured using a respiration ability analyzer. **b** The phase images of fibroblasts (HF) (scale bars = 100 μm) and iPSCs at day 2 (scale bars = 250 μm) and day 6 (scale bars =100 μm) after passage from PCOS patient-derived (PCOS1) and non-PCOS patient-derived iPSCs (non-P1). The cell border and surface area of iPSCs are different. **c** iPSC colonies stained positive for OCT4, SOX2, and NANOG expression. Nuclei were stained with Hoechst 33,342 (blue). Fibroblasts (HF) were stained as negative control. Scale bars = 25 μm. **d** Alkaline phosphatase (ALP) staining and immunofluorescence staining of SSEA-1. Fibroblasts (HF) were stained as negative control. Scale bars = 100 μm. **e** Total and endogenous (Endo) expression levels of *OCT4*, *SOX2*, and *C-MYC* by RT-PCR analysis in PCOS and non-PCOS patient-derived iPSCs, and HF. *GAPDH* was the positive control. **f** G-banding of PCOS and non-PCOS patient derived-iPSCs at passage 10 showed a normal karyotype. **g** H&E staining of teratoma sections of PCOS and non-PCOS patient-derived iPSCs. Neural epithelium and melanocyte epithelium (ectoderm), cartilage (mesoderm), and gut epithelium (endoderm) are shown. Scale bars = 100 μm

Skin fibroblast cells from PCOS patients were collected as adult cells for reprogramming after consent. The epithelial cells were transduced with lentiviral vectors expressing the pluripotency factors *OCT4*, *SOX2*, *KLF4*, and *C-MYC*. iPSC-like colonies were observed 15 to 20 days after viral vector transduction. The morphological specificities of iPSCs include compact colonies, clear boundaries, high nucleus to cytoplasm ratios, and prominent nucleoli. These iPSC colonies were cultured and expanded on MEFs (Fig. 1a). At the same time, non-PCOS patient-derived iPSC lines were established as controls. There were three patients in each group.

The proliferation speed of the PCOS patient-derived iPSCs was lower than that of the non-PCOS patient-derived iPSCs, the area of PCOS patient-derived iPSC colonies was obviously smaller than that of the non-PCOS patient-derived iPSC colonies, and the cell border of PCOS patient-derived iPSCs was not distinct compared with that of non-PCOS patient-derived iPSCs (Fig. 1b and Additional file 2: Figure S1A).

To characterize the iPSCs, we analyzed their pluripotency and differentiation capacities. Immunofluorescence staining revealed the presence of cells expressing the pluripotent-specific surface antigens OCT4, SOX2, and NANOG (Fig. 1c). All iPSCs were positive for ALP activity (Fig. 1d). The expression levels of the total and endogenous pluripotent markers *OCT4*, *SOX2*, and *C-MYC* were detected by RT-PCR, and the exogenous expression of these genes was silenced significantly after 20 passages (Fig. 1e). Karyotype analysis showed that all iPSCs contained normal chromosomes (Fig. 1f). As a hallmark of pluripotency, teratoma formation of iPSC clones was performed (Fig. 1g). Another two lineages from all iPSCs clones were shown (Additional file 2: Figure S1B–E).

### Differential expression of genes between PCOS patient-derived iPSCs and non-PCOS patient-derived iPSCs

The A_260_/A_280_ value of the extracted total RNA was greater than 1.8, and the ratio of the 28S to 18S rRNA, as determined by agarose formaldehyde denaturing gel electrophoresis, was greater than 2. This is the standard of sufficient quality for total RNA with microarray hybridization.

**Fig. 2:**
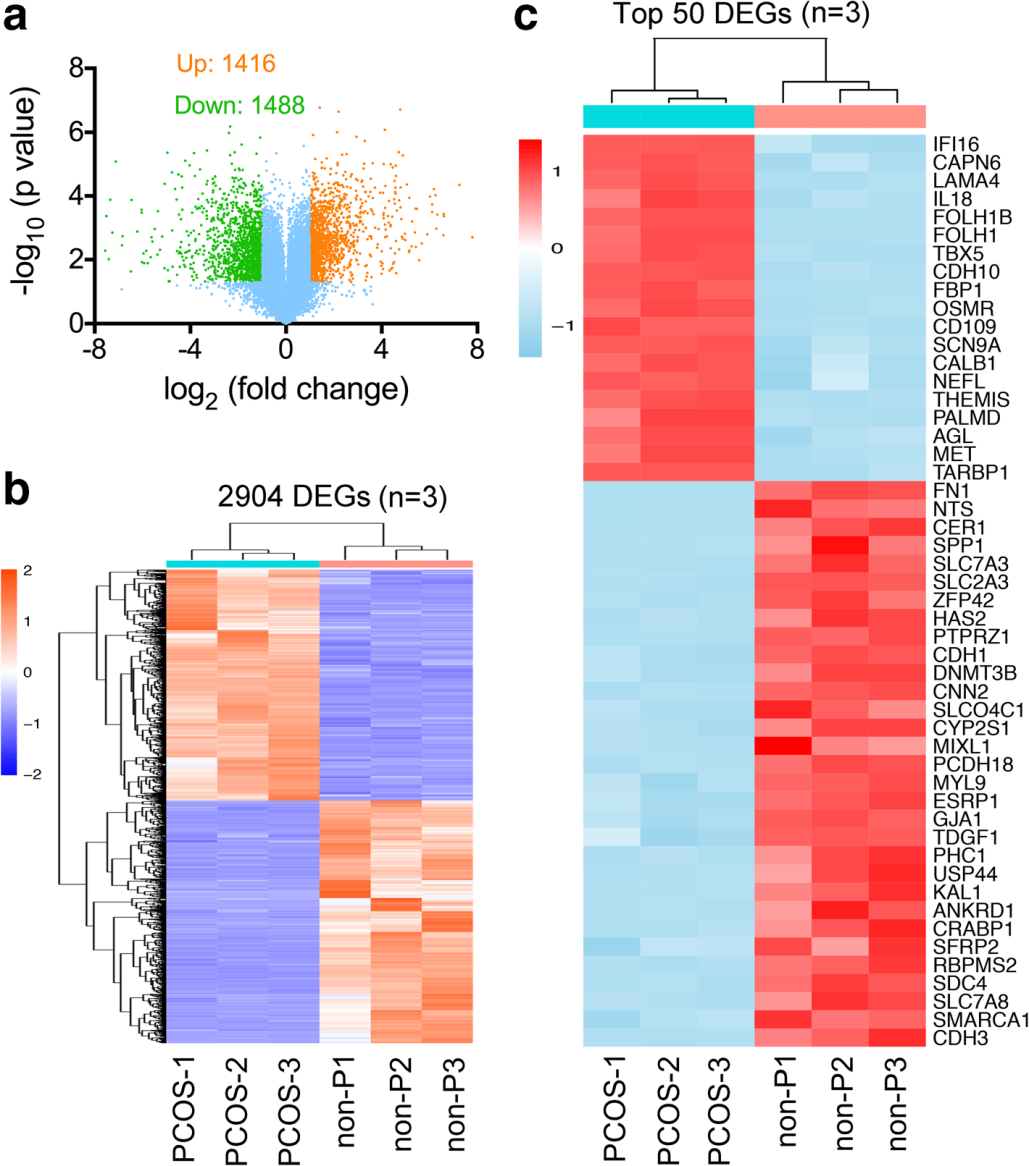
Global differentially expressed genes in RNA microarray analysis. **a** Volcano plot analysis showing significantly altered genes (*q* value < 0.05, fold-change > 2) between polycystic ovary syndrome (PCOS)- and non-PCOS (non-P)-derived iPSCs; 1416 genes were upregulated (orange) and 1488 genes were downregulated (green). **b** Heatmap of differentially expressed genes (2904 probes) between PCOS and non-PCOS expression profiles. Three lines of each group were analyzed (*n* = 3). **c** Heatmap of the top 50 differentially expressed genes (DEGs) between PCOS and non-PCOS expression profiles. Three lines of each group were analyzed (*n* = 3)

To investigate genes that were differentially expressed between PCOS patient-derived and non-PCOS patient-derived iPSCs, we conducted RNA microarray hybridization. Positive signals were obtained from 25,995 clones hybridized with probes. A total of 2904 genes were differentially expressed, of which 1416 genes were upregulated and 1488 genes were downregulated (FC > 2, *p* < 0.01) (Fig. 2a, b).

### Analysis of microarray expression files in PCOS patient-derived iPSCs

**Fig. 3:**
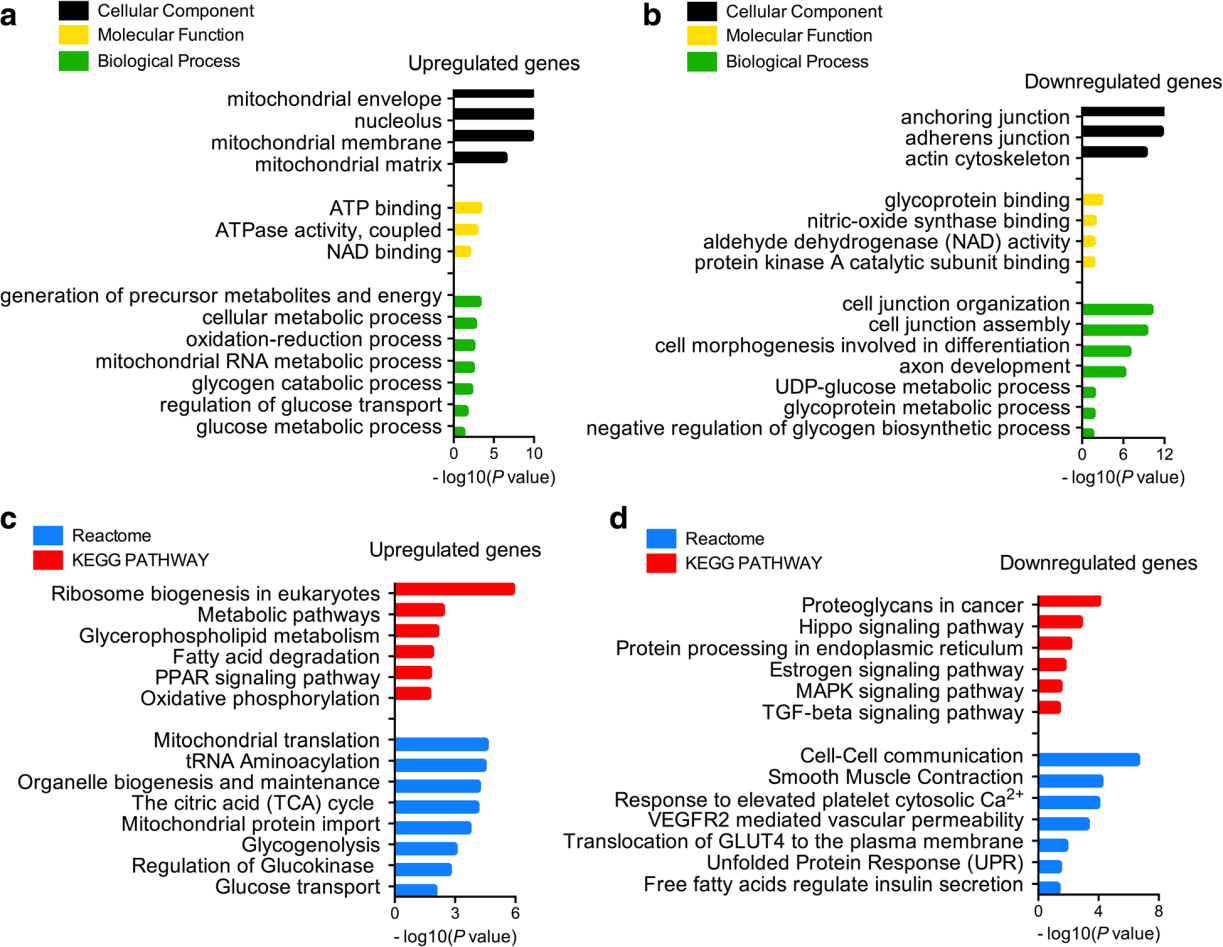
Enrichment analysis of differentially regulated genes in PCOS- and non-PCOS patient-derived iPSCs. **a**, **b** GO terms based on cellular_component, molecular_ function, and biological_process of the upregulated and downregulated genes. **c**, **d** The up- and downregulated genes were enriched by Ractome and KEGG database

The transcripts between PCOS patient-derived and non-PCOS patient-derived iPSCs were analyzed using different software and statistical tools. Differentially expressed genes (DEGs) between PCOS patient-derived and non-PCOS patient-derived iPSCs were screened using the lima package followed by exclusion of DEGs with the genefilter package. Enrichment analysis was performed on DEGs using AmiGO2. The top 10 DEGs associated with known PCOS characteristics are summarized in Table 2 and Table 3. The top 50 DEGs were shown in a heatmap (Fig. 2c). GO term enrichment showed that upregulated genes were enriched in metabolic processes and mitochondrial activities, which participated in the TCA cycle, the respiratory electron transport chain (ETC), and glycogenolysis (Fig. 3a). On the other hand, the downregulated genes were related to cell communication, glucose transport, and uptake (Fig. 3b). Besides, these up- and downregulated genes were enriched with KEGG pathway and Reactome database (Fig. 3c, d).

**Table 2:** The top 10 significantly downregulated genes in polycystic ovary syndrome patient-derived induced pluripotent stem cells

Gene name	Gene number	Gene Ontology molecular function term	Fold-change
FN1	NM_002026	Cell adhesion, protein binding, inflammatory response	0.001
NTS	NM_006183	Neuropeptide hormone activity, signal transduction	0.002
CER1	NM_005454	Cytokine activity, morphogen activity, BMP binding	0.002
SPP1	NM_000582	Cytokine activity, ossification, transforming growth factor (TGF)-β signaling	0.003
SLC7A3	NM_001048164	Amino acid and ion transport, transmembrane transport	0.003
SLC2A3	NM_006931	Glucose transmembrane transporter activity	0.004
ZFP42	NM_174900	Sequence-specific DNA binding transcription activity,	0.004
HAS2	NM_005328	Hyaluronan synthase activity, kidney development	0.005
PTPRZ1	NM_001206838	Protein dephosphorylation, axonogenesis	0.005
CDH1	NM_004360	Calcium ion binding, protein phosphatase binding	0.006

**Table 3:** The top 10 significantly upregulated genes in polycystic ovary syndrome patient-derived induced pluripotent stem cells

Gene name	Gene number	Gene Ontology molecular function term	Fold-change
IFI16	NM_001206567	Transcription, cell proliferation, hemopoiesis	1484
CAPN6	NM_014289	Calcium-dependent cysteine-type endopeptidase	221.7
LAMA4	NM_001105206	Extracellular matrix structural constituent	151.6
IL18	NM_001562	MAPK cascade, inflammatory and immune response	96.9
FOLH1B	NM_153696	Metallopeptidase and dipeptidase activity, proteolysis	80.2
FOLH1	NM_004476	Folic acid-containing compound metabolic process	78.2
TBX5	NM_080718	Transcription factor activity, heart development	76.4
FBP1	NM_000507	AMP binding, fructose and glucose metabolic process	69.2
AGL	NM_000028	Glycogen debranching enzyme, glucose metabolism	42.7
KIAA1324	NM_020775	Macroautophagy, regulation of apoptosis, trans-Golgi	29.6

### Verification of differentially expressed genes by RT-PCR

**Fig. 4:**
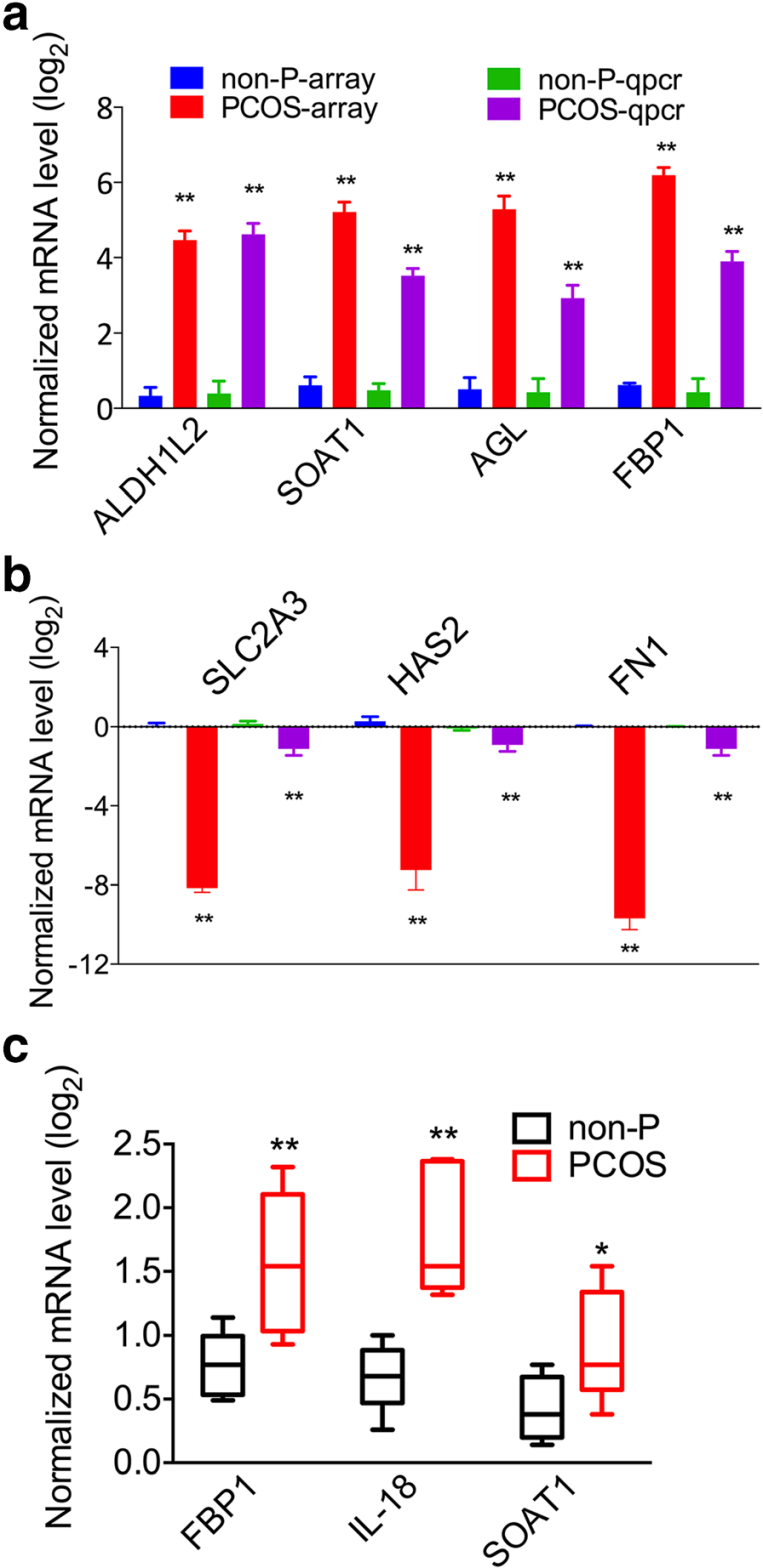
RT-PCR verification of microarray results. **a**, **b** Upregulated and downregulated gene fold-changes in the RNA microarrays and quantitative polymerase chain reaction (qpcr). **c** Upregulated genes were verified by RT-PCR in granulosa cells of other five polycystic ovary syndrome (PCOS) patients and five non-PCOS (non-P) patients (*n* = 5). Error bars represent SD; *P < 0.05, **P < 0.01, versus non-P

To validate the microarray data, some DEGs were selected for quantitative RT-PCR analysis. These genes were dysregulated in PCOS patient-derived iPSCs and were involved in glycogen metabolism and glycolysis. The expression levels of selected genes (*AGL*, *FBP1*, *SLC2A3*, *FN1* etc.) were similar in RNA microarray and RT-PCR analysis (Fig. 4a, b), although the absolute ratios were different due to the potential differences in assay sensitivity and dynamics between the two methods. Furthermore, the DEGs were verified by RT-PCR in the granulosa cells of other five PCOS and five non-PCOS patients, and these performed consistent with changes observed in PCOS patient-derived iPSCs (Fig. 4c).

### Mitochondrial respiration ability and glycolysis function in PCOS patient-derived iPSCs

**Fig. 5:**
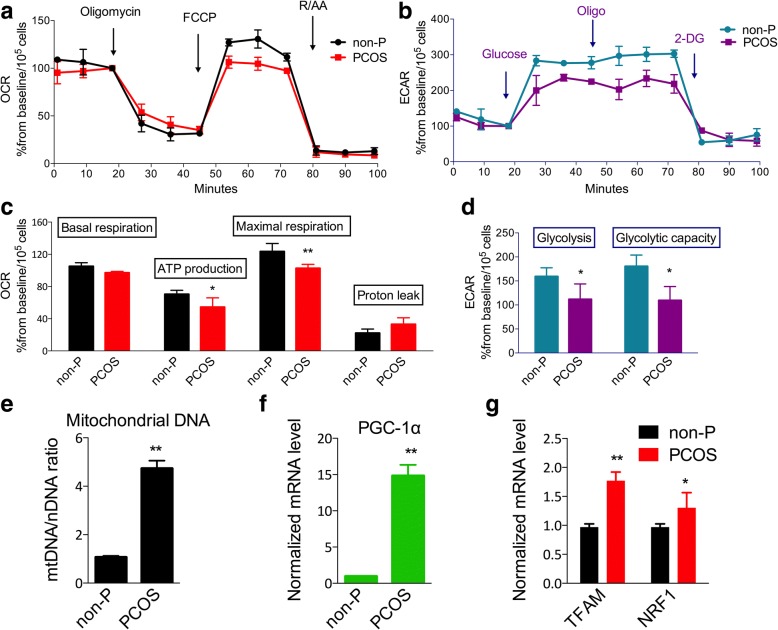
Mitochondrial respiration ability and glycolysis function in polycystic ovary syndrome (PCOS) patient-derived iPSCs. **a** Mitochondrial function based on the in-vitro oxygen consumption rate (OCR) in response to oligomycin, FCCP, rotenone, and antimycin (R/AA). **b** Glycolysis function based on the extracellular acidification rate (ECAR) in response to glucose, oligomycin, and 2-DG. **c** Quantitative analysis of basal oxygen consumption, ATP production, maximal respiration, and proton leak of iPSCs. **d** Quantitative analysis of glycolysis and glycolytic capacity of iPSCs. **e** Mitochondrial DNA (mtDNA) numbers normalized to nuclear DNA (nDNA). **f** Expression of mitochondrial RNA biogenesis gene *PGC-1α*. **g** Expression of mitochondrial RNA biogenesis related genes *TFAM* and *NRF1*. *n* = 3; error bars represent SD; *P < 0.05, **P < 0.01, versus non-PCOS (non-P)

The enriched GO terms mainly focused on mitochondrial components and glucose metabolic processes. To determine the mitochondrial function of iPSCs, the mitochondrial respiration abilities of PCOS patient-derived and non-PCOS patient-derived iPSCs were measured. The basal respiration rate was little attenuated in PCOS patient-derived iPSCs. However, oligomycin, an ATP synthase inhibitor, led to comparable inhibition of respiration in both PCOS patient-derived and non-PCOS patient-derived iPSCs. Maximal respiration ability, assessed by the addition of the uncoupler FCCP, was also significantly decreased in PCOS patient-derived iPSCs. The cytochrome c oxidase (complex IV) inhibitor RA led to inhibition of respiration in both iPSC populations. The defective respiration ability because of the mitochondrial dysfunction was measured in PCOS patient-derived iPSCs (Fig. 5a, c). At the same time, the glycolysis function of iPSCs was measured by similar methods to mitochondrial function. The results of glycolysis function measurements also showed decreased glycolysis ability and inhibited maximal glycolytic capacity in PCOS patient-derived iPSCs compared with normal iPSCs (Fig. 5b, d).

### Mitochondria biogenesis in PCOS patient-derived iPSCs

To investigate the mechanism of mitochondrial defects in PCOS patient-derived iPSCs, mitochondrial biogenesis was evaluated in iPSCs. First, mtDNA content in PCOS and non-PCOS patient-derived iPSCs was measured by quantitative RT-PCR. The mtDNA content was calculated by measuring the ratio of mtDNA to β-actin (nuclear gene). Unexpectedly, the mtDNA content in PCOS patient-derived iPSCs was significantly higher than that of non-PCOS patient-derived iPSCs (Fig. 5e). Furthermore, we measured the mRNA levels of genes implicated in mitochondrial biogenesis, such as peroxisome proliferator-activated receptor gamma coactivator 1 alpha (PGC-1α) and mitochondrial transcription factor A (TFAM). Consistent with increased mtDNA content, the *PGC-1α*, *TFAM*, and *NRF1* expression levels were greater in PCOS patient-derived iPSCs compared with non-PCOS patient-derived iPSCs (Fig. 5f, g). Together, these data demonstrated a mitochondrial biogenic response in PCOS patient-derived iPSCs.

### Metformin effects on PCOS patient-derived iPSCs

**Fig. 6:**
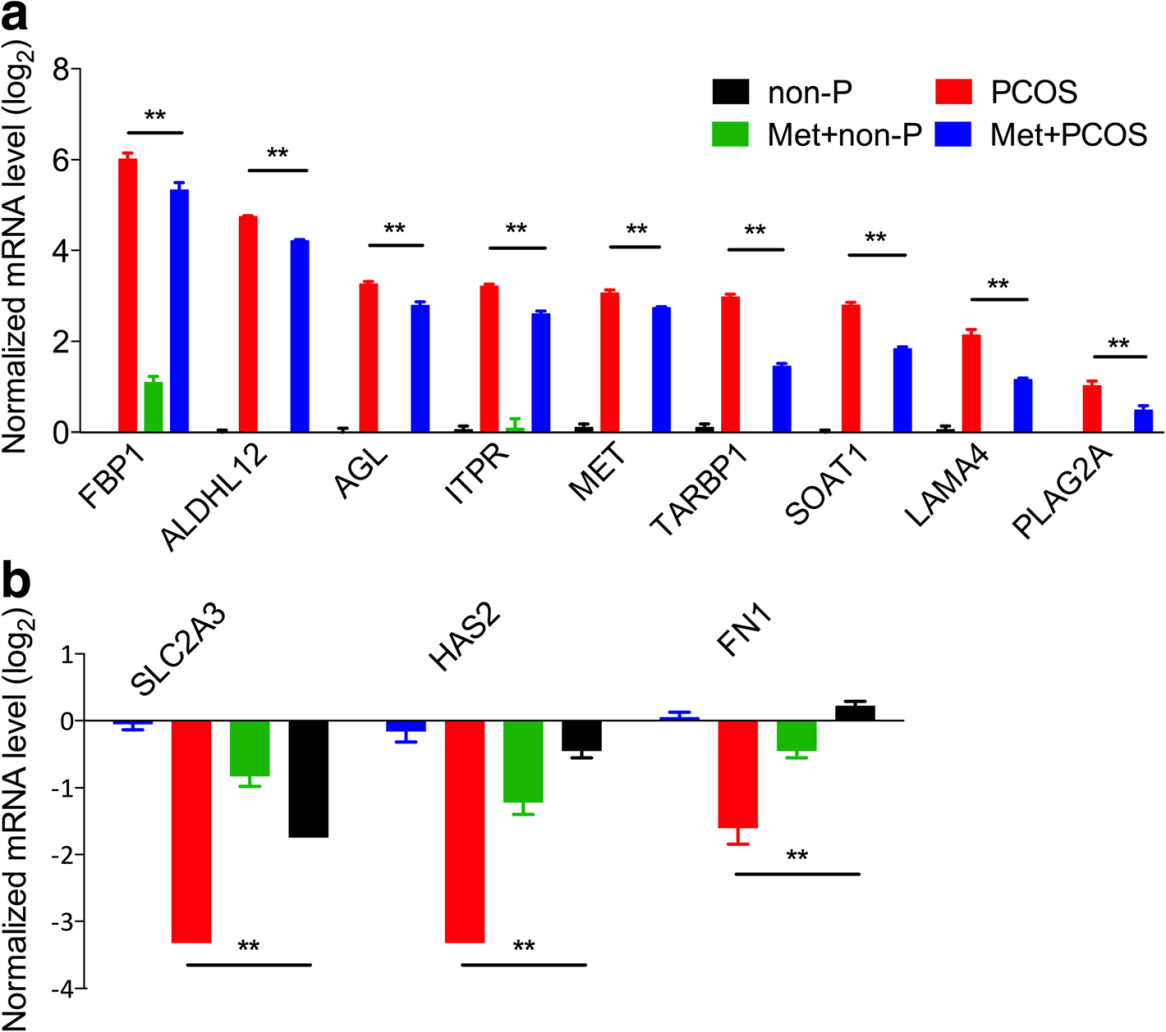
Metformin effects on dysregulated genes in PCOS patient-derived iPSCs. **a** Upregulated genes were increased after treatment with metformin (Met; 1 mM) for 24 h in polycystic ovary syndrome (PCOS) patient-derived iPSCs compared with non-PCOS (non-P) patient-derived iPSCs. **b** Downregulated genes were decreased in PCOS patient-derived iPSCs compared with non-PCOS patient-derived iPSCs. *n* = 3; error bars represent SD; *P < 0.05, *P < 0.01

**Fig. 7:**
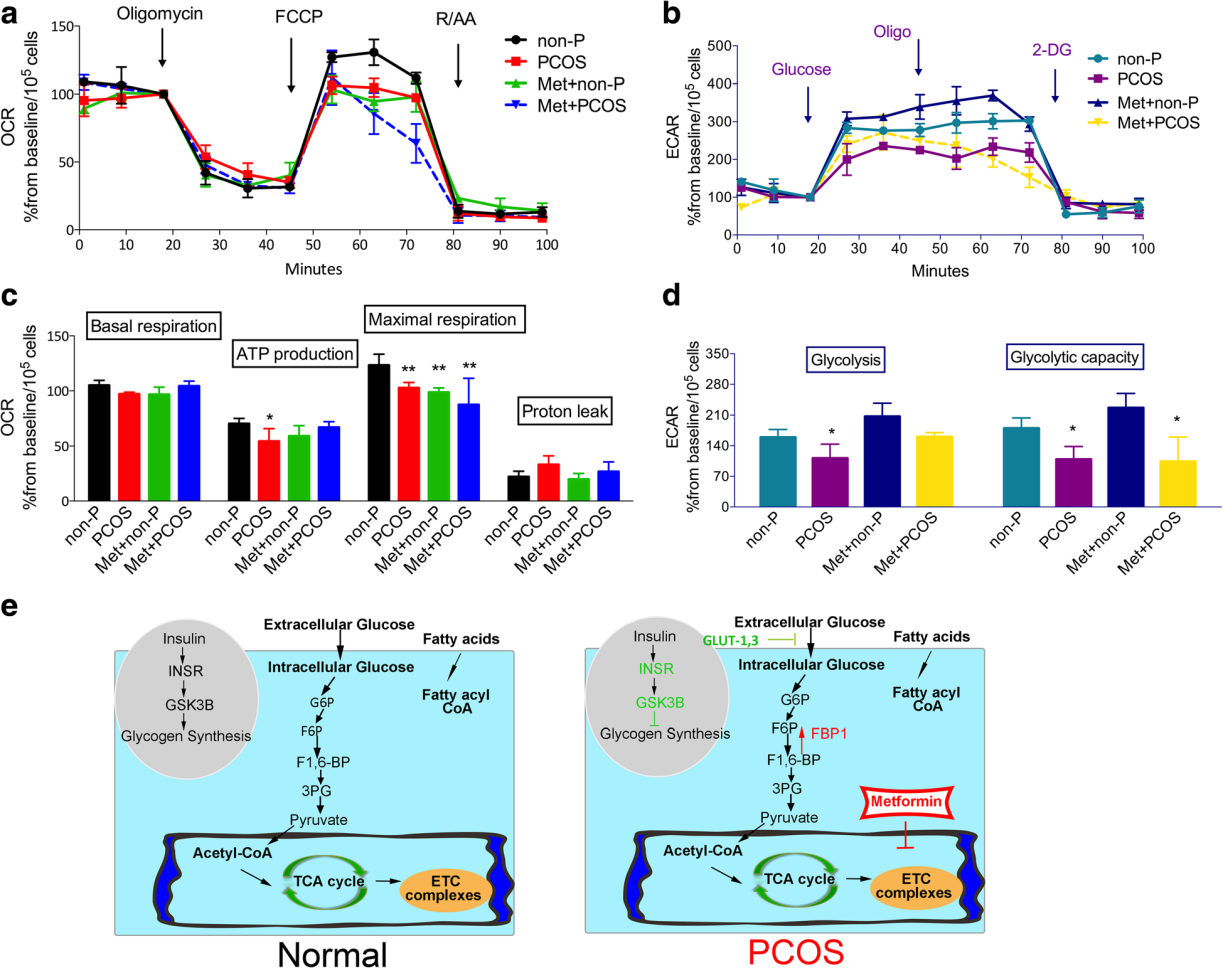
Metformin effects on mitochondrial respiration ability and glycolysis function of PCOS patient-derived iPSCs. **a** Mitochondrial respiration function in polycystic ovary syndrome (PCOS) patient-derived iPSCs after treatment with metformin (Met). **b** Glycolysis function in PCOS patient-derived iPSCs after treatment with metformin. **c**, **d** Quantitative analysis of mitochondrial ability and glycolysis function in PCOS patient-derived iPSCs treated with metformin. *n* = 3; error bars represent SD; *P < 0.05, *P < 0.01, versus non-PCOS (non-P). **e** Conclusion of mitochondrial dysfunction and metabolism abnormality in PCOS. Red: upregulated gene in PCOS patient-derived iPSCs; green: downregulated gene in PCOS patient-derived iPSCs. ECAR extracellular acidification rate, ETC electron transport chain, OCR oxygen consumption rate, R/AA rotenone and antimycin, TCA tricarboxylic acid

The biguanide metformin is a potent antihyperglycemic agent used to treat type II diabetes that is also effective in PCOS treatment. Metformin can improve fertility in PCOS patients and effectively decreases cardiovascular complications. Numerous studies have linked metformin therapy with the activation of AMP-activated protein kinase (AMPK), which is involved in the inhibition of mitochondrial complex I. Metformin treatment can partially rescue the dysregulated genes in PCOS patient-derived iPSCs compared with non-PCOS patient-derived iPSCs (Fig. 6a, b). These genes are mainly involved in glycogenolysis, gluconeogenesis, and some other metabolic processes. The ATP production ability and glycolysis ability of PCOS patient-derived iPSCs were also partially returned to normal levels. Despite this, metformin had little effect on mitochondrial maximal respiration ability and maximal glycolytic capacity (Fig. 7a–d). Metformin can stimulate glycolysis through activation of glycolytic enzyme. Meanwhile, metformin can possibly inhibit mitochondrial complex I and IV, decreasing ATP production and inhibiting gluconeogenesis [30]. Therefore, PCOS patient-derived iPSCs treated with metformin could have lower respiration ability compared with those in non-PCOS patient-derived iPSCs.

## Discussion

The iPSC lines in this study were generated from PCOS patients and were similar to human embryonic stem cells (ESCs) in many respects, including morphology and expression of pluripotency-associated genes. Furthermore, we analyzed the expression profiles of PCOS patient-derived iPSCs using RNA microarray and studied the mitochondrial function in these cells. To the best of our knowledge, this is the first functional study of PCOS patient-derived iPSCs. The results obtained here provide a new approach for future explorations of the molecular mechanisms of PCOS and for developing new therapies for PCOS.

PCOS is considered a polygenic pathology with interaction of susceptible genomic variants and environmental factors. The metabolic characteristics in this study only represent diagnosed PCOS patients, and cannot be extrapolated to other kinds of PCOS patients. The pathological phenotypes associated with PCOS differ among different populations. The multiplicity of PCOS pathogens during this study might be a limitation. The limitation for PCOS iPSC generation is heterogeneity of PCOS. Although the three selected PCOS patients were of the severe and classic type, the small number of patients limits the generalizability of these findings to all patients with PCOS. In addition, these results are exploratory and hypothesis-generating findings that need to be replicated in other cohorts, especially in clinical specimens of PCOS patients.

A variety of animal models for PCOS have been generated through increased androgen exposure, containing PCOS-like reproductive and metabolic traits in female rodents and nonhuman primates [31, 32]. The PCOS models using animals only represent PCOS-like phenotypes since the pathogenesis of human PCOS is complex. For example, animal models were only established by a single induction, instead of a natural disease occurring and developing. Moreover, the genetic differences between animals and humans makes the use of animal models for human PCOS more difficult. Therefore, iPSCs derived from PCOS patients have recognized potential for the generation of a disease model.

Using a four-factor lentiviral system, we generated iPSCs from PCOS and healthy fibroblasts. Interestingly, we observed significant phenotypic differences between PCOS patient-derived and non-PCOS patient-derived iPSCs, including proliferation speed, colony area, and cell border appearance. PCOS patient-derived iPSCs always showed much slower proliferation rates than non-PCOS patient-derived iPSCs, which may have resulted from distinct differences in the cell cycle, metabolism, and apoptosis.

The differentially expressed genes identified from the microarray profiles included genes associated with glycometabolism, lipid metabolism, and steroid hormones, which are also associated with classic PCOS characteristics. Fructose-1,6-bisphosphatase (FBP1) was upregulated in PCOS iPSCs, which was related to gluconeogenesis and produced redundant glucose in PCOS cells. The transcription factor RUNX2 was found to be upregulated in PCOS iPSCs, which was recently reported to be upregulated by luteinizing hormone (LH) both in rats and humans [33]. There were other genes related to cardiovascular disease which were associated with PCOS.

Facilitative glucose transporters (GLUTs) are necessary for glucose transport activities in cells. Glucose limitation related to GLUT1 deficiency has been reported to result in decreased mitochondrial function, such as decreased mitochondrial membrane potential and activation of mitochondrial-dependent apoptosis. Both the microarray profile and RT-PCR results showed that the expression of GLUT1 and GLUT3 were decreased in PCOS patient-derived iPSCs. Therefore, decreased expression of GLUTs may result in downregulation of glucose uptake with IR in PCOS (Fig. 7e).

Mitochondrial function is critical for cellular energy production via the glycolysis and TCA cycle pathways. The mitochondria generate most of the cell’s supply of ATP through the ETC. However, the only source of citrate in the cell is the mitochondrial TCA cycle. Mitochondria are the major reactive oxygen species (ROS) generators as well as a main target of ROS-induced oxidative damage. Given the alterations in mitochondrial content and biogenesis, we tested whether mitochondrial function and glucose metabolism were disrupted in PCOS patient-derived iPSCs. We measured the mitochondrial respiration rate and glycolysis function of PCOS patient-derived iPSCs using the XF Seahorse analyzer. The downregulation of mitochondrial respiration ability and glycolysis function in PCOS patient-derived iPSCs indicated the cause of metabolism defects in PCOS. The mitochondria are regulated through a balance between fission and fusion events. Unexpectedly, the mitochondrial content and biogenesis were increased in PCOS patient-derived iPSCs. These phenomena indicated that the integrity of mitochondria was affected in PCOS patient-derived iPSCs, leading to mitochondrial dysfunction. The decrease in mitochondrial function may then stimulate more mitochondrial biogenesis by a compensatory effect [18].

## Conclusion

In conclusion, this study has identified a number of PCOS-associated genes that may contribute to the functional effects on PCOS. The microarray analysis and mitochondrial ability measures showed metabolic disorder and mitochondrial dysfunction of PCOS patient-derived iPSCs, indicating that iPSCs can be derived from PCOS patients. PCOS patient-derived iPSCs can thus potentially be used for disease modeling in vitro to improve our understanding and diagnosis of PCOS, and to help drug discovery for PCOS.

## Additional files

Additional file 1: **Table S1.** The quantitative PCR primers used in this study. (DOC 36 kb)

Additional file 2: **Figure S1.** The characteristics of PCOS patient-derived iPSCs and non-PCOS patient-derived iPSCs. (TIF 155301 kb)
