# Supplementary material for: New insights into the genic and metabolic characteristics of induced pluripotent stem cells from polycystic ovary syndrome women
Source: Stem Cell Res Ther. 2018 Aug 9;9:210. doi: 10.1186/s13287-018-0950-x (PMC6085636; doi:10.1186/s13287-018-0950-x)
Supplement: Supplementary file 1 — Table S1. The quantitative PCR primers used in this study. (DOC 36 kb) [file 13287_2018_950_MOESM1_ESM.doc]

**Supplementary Table S1** Primers used in the study.

| Gene | Forward primers (5'-3') | Reverse primers (5'-3') |
| --- | --- | --- |
| *GLUT3* | CTTCACCGGCTTCCTCATTAC | GCCGTCCTTTCCAGATCTATC |
| *HAS2* | CTGTCCAGCTAGTAGGTCTCA | GCGGGAAGTAAACTCGACAT |
| *FN1* | GATGCTACTGAGACCACCATC | GGTGTAGCTTCTGACATCTGG |
| *FBP1* | CTGCCGTCACTGAGTACATC | ATATCCCTCCGTAGACCAGAG |
| *AGL* | GCCTAGCTGTTGACAATGCAG | GTATGTCATAGCACCCTCCAC |
| *ALDH1L2* | ATCCGCAAACTTGGTTTCAC | GCACAGCCTTGTCAAGTTCA |
| *LAMA4* | GGGATGCCGAAGACATGAAC | AGGTGTTGTCAGAGAGTCCG |
| *SOAT1* | GTTACGCTTTGGTGACAGGA | CAGCTAACATGGCAGCAGAT |
| *IL18* | ATTGACCAAGGAAATCGGCC | CCTCTAGGCTGGCTATCTTT |
| *PLA2G4A* | GGCTGAAGGAGTGCTATGTC | CTCTTCCTCAGTTTCCCTTGG |
| *OCT4*-total | CCCCAGGGCCCCATTTTGGTACC | ACCTCAGTTTGAATGCATGGGAGAGC |
| *SOX2-*total | TTCACATGTCCCAGCACTACCAGA | TCACATGTGTGAGAGGGGCAGTGTGC |
| *OCT4*-Endo | CCTTCGCAAGCCCTCATTTCAC | CATCGGAGTTGCTCTCCACC |
| *SOX2-*Endo | TCAGGAGTTGTCAAGGCAGAG | TCCGGGCTGTTTTTCTGGTT |
